# Systematization of Oncoplastic Surgery: Selection of Surgical Techniques and Patient-Reported Outcome in a Cohort of 1,035 Patients

**DOI:** 10.1245/s10434-015-4396-4

**Published:** 2015-02-12

**Authors:** Mahdi Rezai, Sarah Knispel, Stephanie Kellersmann, Hildegard Lax, Rainer Kimmig, Peter Kern

**Affiliations:** 1 Breast Unit, Breast Center Düsseldorf Luisenkrankenhaus, Düsseldorf, Germany; 2 Women’s Department, University Hospital of Essen, Essen, Germany; 4 Institute of Medical Informatics, Biometry and Epidemiology, University of Duisburg-Essen, Essen, Germany

## Abstract

**Introduction:**

Functional and aesthetic outcome after breast-conserving surgery are vital endpoints for patients with primary breast cancer. A large variety of oncoplastic techniques exist; however, it remains unclear which techniques yield the highest rates of local control at first surgery, omission of reexcision or subsequent mastectomy, and merits the highest degree of patient satisfaction.

**Methods:**

In this retrospective case cohort trial with a customized investigational questionnaire for assessment of patient satisfaction with the surgical result, we analyzed 1,035 patients with primary, unilateral breast cancer and oncoplastic surgery from 2004 to 2009.

**Results:**

Analysis of patient reported outcome (PRO) revealed that 88 % of the cohort was satisfied with their aesthetic result using oncoplastic techniques following the concept presented. These results also were achieved in difficult tumor localizations, such as upper inner and lower inner quadrant. Conversion rate from breast-conserving therapy to secondary mastectomy was low at 7.2 % (*n* = 68/944 patients). The systematization of oncoplastic techniques presented—embedded in a multimodal concept of breast cancer therapy—facilitates tumor control with a few number of uncomplicated techniques adapted to tumor site and size with a median resection of 32 (range 11–793) g. Five-year recurrence rate in our cohort was 4.0 %.

**Conclusions:**

Patient´s satisfaction was independent from age, body mass index, resection volume, tumor localization, and type of oncoplastic surgery (*p* > 0.05). We identified postoperative *pain* as an important negative impact factor on patient´s satisfaction with the aesthetic result (*p* = 0.0001).

**Electronic supplementary material:**

The online version of this article (doi:10.1245/s10434-015-4396-4) contains supplementary material, which is available to authorized users.

The oncologic outcome of breast-conserving surgery is equivalent to mastectomy, when free margins are achieved and adjuvant radiotherapy of the operated breast is applied.^1^–^5^ Oncoplastic breast conserving techniques combine two aspects: oncological safety with a resection of the tumor with free margins and optimal aesthetic aspects.^6^–^8^ Breast-conserving oncoplastic techniques divide into volume displacement and volume replacement techniques: the first are constituted by rotational mammaplasty techniques (glandular rotation mammaplasty, dermoglandular rotation mammaplasty and tumor-adapted mastopexy), the latter by latissimus-dorsi-flap and lateral thoracic advancement flap.^9^–^11^ We investigated the options and limitations of oncoplastic surgery as well as patient satisfaction in a large cohort of oncoplastic patients. As primary endpoints, we defined: the oncological safety of oncoplastic breast surgery (clear margins, low recurrence rate) and feasibility (reexcision rates, secondary mastectomy rates) as well as patient satisfaction [patient-reported outcome (PRO)].

## Patients and Methods

We analysed data of 1,035 oncoplastic patients in a breast unit of maximum care from 2004 to 2009 retrieved from patient charts and used a customized questionnaire for evaluation of current patient satisfaction. An additional questionnaire as a validated instrument of perceived esthetic and functional status of the breast was used, i.e., “BCTOS” (Breast Cancer Treatment Outcome Scale), first described by Stanton et al.,^12^ and ratings in this scale were correlated to the ratings in our customized questionnaire.

Data cutoff was at February 2013. We explored the following characteristics, comorbidities, and surgery-related complications:

- Patient characteristics (body-mass-index, age, menarche, menopause, family history of breast cancer)
- Tumor characteristics (histology, TNM-classification, immunohistochemical subtype, tumor localization)
- Surgical treatment characteristics (local therapy: operation—type of surgery, margins, reexcision rate, resection volume)
- Physical sequelae/complications: (early: <14 days; late: ≥14 days)
- Pain scale
- Secondary mastectomy rates and its influencing factors
- Patient satisfaction with the aesthetic outcome
- Disease-free and overall survival

Intrinsic subtypes have been approximated by immunohistochemical characterization according to 12th St. Gallen International Consensus Conference.^13^ This study complies with the principles of the Declaration of Helsinki and was approved by the institutional review board.

### Surgical Techniques

Surgical techniques were based first on tumour location, second on the condition whether the lesion was unicentric or multicentric, and whether resection volume would exceed >20 % of the breast. For all locations of the upper hemisphere of the breast and unicentric tumours, glandular rotation mammaplasty was the standard option for reshaping of the breast. With multicentricity or breast resection >20 % or tumours of the lower hemisphere of the breast, a reduction mammaplasty pattern was applied (inferior-pedicled technique described by Ribeiro in the modification of the author) to reconstitute the optimal breast form. This procedure avoids birds peak deformations for patients with gross resection of tissue in the lower quadrants of the breast. Where fat tissue was readily accessible for volume displacement without necessity of musculocutaneous flaps, this was incorporated in the concept of reshaping of the breast such as the thoracoepigastric flap for the lower quadrants (in cases of skin resection) and lateral thoracic advancement flap for the upper outer quadrant (in cases with need of additional volume replacement).^9^

### Statistics

**Fig. 1:**
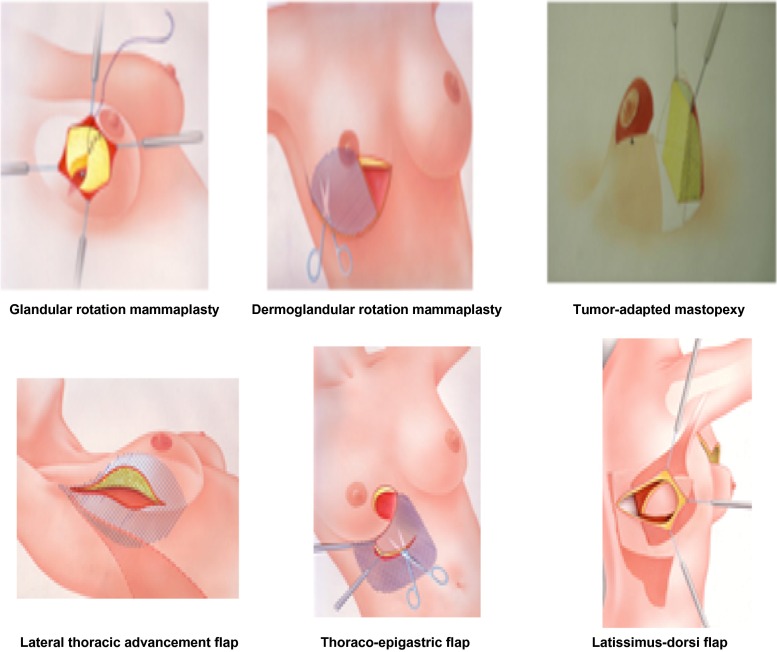
Surgical techniques

Because statistical tests—*χ* ^2^/Likelihood, Mantel–Haenszel test, and Wilcoxon’s test—for calculation were applied, *p* values must be seen as descriptive, not adjusted. Log-rank test and Wilcoxon rank-sum tests were used (Fig. 1). To calculate 5-year overall survival and disease-free survival, the date of death or local recurrence was defined as the endpoint, respectively, and the duration of follow-up was calculated as the date of breast-conserving surgery to this endpoint.

## Results

Of 1,035 patients with oncoplastic operations, 944 patients met the inclusion criteria (REMARK diagram, see Supplement, Material 1); 70.7 % (624/882) of patients responded to the emission of questionnaires. Average age was 57.6 years (median 58, range 25–88 years). Patient characteristics are shown in Table 1.

**Table 1:** Patient characteristics

	Cohort Prozent		Responders		Nonresponders	
	*n* = 944		*n* = 624		*n* = 320	
Characteristics	No.	%	No.	%	No.	%
Age group at time of surgery (year)						
20–29	7	0.7	1	0.2	6	1.9
30–39	41	4.3	21	3.4	20	6.3
40–49	190	20.1	110	17.6	80	25.0
50–59	261	27.7	172	27.5	89	27.7
60–69	305	32.3	215	34.5	90	28.1
70–79	128	13.6	100	16.0	28	8.8
80–89	12	1.3	5	0.8	7	2.2
Unknown	0	0	0	0.0	0	0.0
BMI						
Underweight (BMI 15–19.9 kg/m^2^)	56	5.9	27	4.3	29	9.0
Normal weight (BMI 20–24.9 kg/m^2^)	534	56.6	356	57.1	178	55.6
Overweight (BMI 25–29.9 kg/m^2^)	269	28.5	184	29.5	85	26.7
Obesity (BMI > 30.0 kg/m^2^)	72	7.6	51	8.2	21	6.6
Unknown	13	1.4	6	0.9	7	2.1
Age of menarche (year)						
<12	70	7.4	68	10.9		
12–16	509	53.9	509	81.6		
17–20	20	2.1	20	3.2		
>20	2	0.2	2	0.3		
Unknown	343	36.3	25	4.0		
Age at menopause (year)						
<30	3	0.3	3	0.5		
30–39	32	3.4	32	5.2		
40–49	239	25.3	239	38.3		
50–59	206	21.8	206	33.0		
≥60	7	0.7	7	1.1		
Unknown	457	48.4	137	21.9		
Menopause status at time of surgery						
Premenopausal	61	6.5	61	9.8	0	0.0
Perimenopausal	11	1.2	11	1.8	0	0.0
Postmenopausal	576	61.8	469	75.2	107	33.4
Unknown	296	31.3	83	13.2	213	66,6
Hormone replacement therapy						
Administered, duration unknown	152	16.1	109	17.5	43	13.5
Administered up to 10 years	123	13.0	120	19.2	3	0.9
Administered 10 years or more	270	28.6	269	43.1	1	0.3
Not administered	121	12.9	114	18.3	7	2.2
Unknown	278	29.4	12	1.9	266	83.1
Family history of breast cancer						
BRCA-positive	18	1.9	18	2.9	0	0.0
BRCA-negative	235	24.5	194	31.1	41	12.8
Negative	398	42.2	397	63.6	1	0.3
Unknown	2934	31.4	15	2.4	278	86.9

**Fig. 2:**
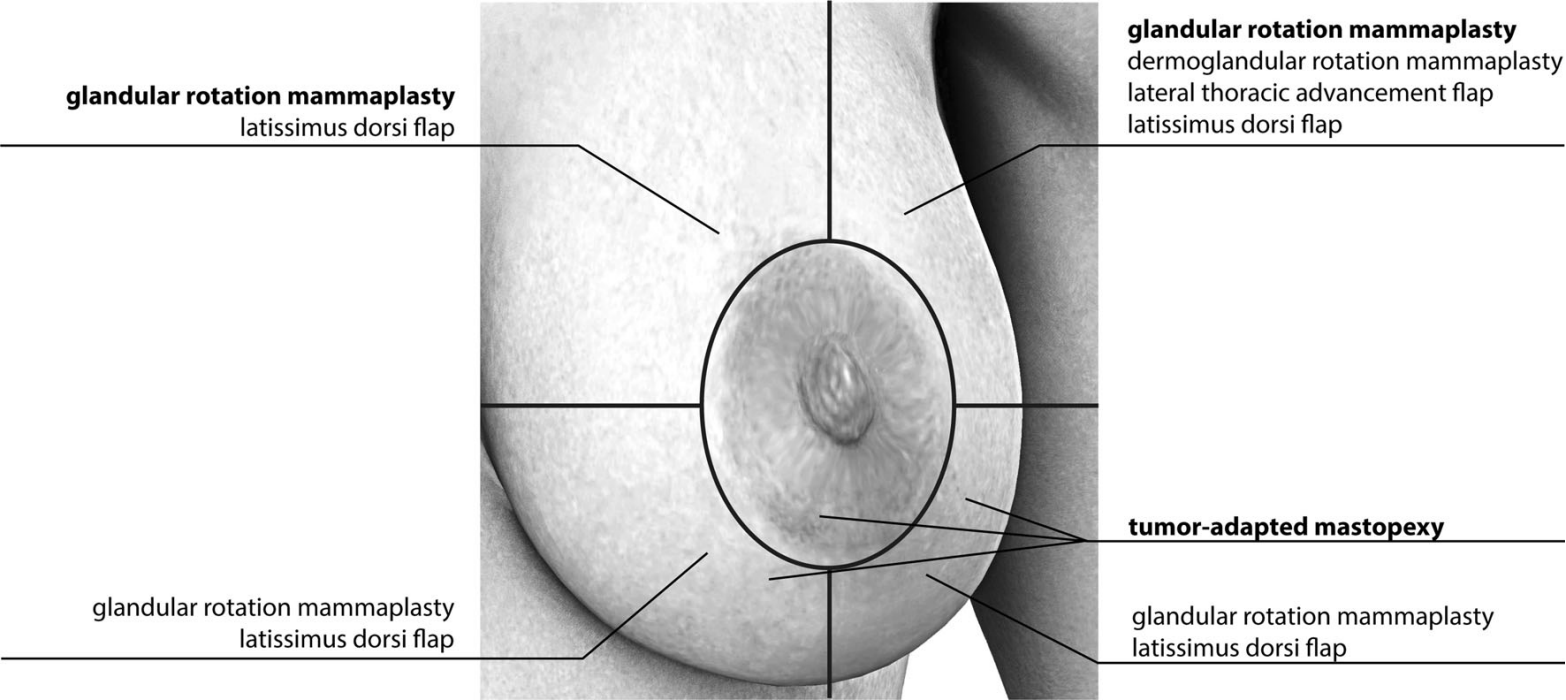
Selection of oncoplastic techniques

The selection of an oncoplastic technique presented follows a nomogram that we adapted to the tumor localization, tumor size, and the volume of the breast. The different choices of techniques are displayed in Fig. 2.

In the lower hemisphere of the breast, 55.4 % of tumors were operated by tumor-adapted mastopexy, whereas glandular rotation mammoplasty was less frequently used here (36.2 %). In the upper part of the breast, glandular rotation mammoplasty was the most frequently used technique (69.5 %), whereas tumor-adapted mastopexy was not commonly performed (11.3 %, *p* > 0.001). One-third (29.7 %) of multicentric or multifocal tumors were operated by tumor-adapted mastopexy. Dermoglandular rotation mammaplasty and lateral thoracic advancement flap are predominantly performed in cases of involvement of the upper outer quadrant of the breast. Glandular rotation mammaplasty and latissimus-dorsi-flap were not associated with specific tumor locations (Table 2). The tumor-adapted mastopexy was characterized by a significantly higher median resection volume (52 g) compared with the glandular rotation mammaplasty (29 g; *p* < 0.001). Tumor size was not a determining factor for the choice of a certain oncoplastic technique (*p* > 0.05).

**Table 2:** Oncoplastic techniques by tumor location

	Tumor location					
Oncoplastic techniques	Lower^a^	Multifocal/multicentric	NAC and horizontal transition^b^	Upper^c^	Unknown	Total
	*n* (%)	*n* (%)	*n* (%)	*n* (%)	*n* (%)	*n* (%)
Glandular rotation mammaplasty	47 (36.2)	70 (63.1)	68 (68.0)	405 (69.5)	12 (60.0)	602 (63.8)
Dermoglandular rotation mammaplasty	8 (6.2)	2 (1.8)	3 (3.0)	49 (8.4)	1 (5.0)	63 (6.7)
Tumor-adapted mastopexy	72 (55.4)	33 (29.7)	23 (23.0)	66 (11.3)	3 (15.0)	197 (20.9)
Lateral thoracic advancement flap	2 (1.5)	1 (0.9)	3 (3.0)	36 (6.2)	0 (0.0)	42 (4.4)
Latissimus-dorsi flap	0	1 (0.9)	0	5 (0.9)	1 (5.0)	7 (0.7)
Others	1 (0.8)	2 (2.7)	3 (3.0)	19 (3.3)	1 (5.0)	27 (2.9)
Unknown	0	1 (0.9)	0	3 (0.5)	2 (10.0)	6 (0.6)
Total	130 (13.8)	111 (11.8)	100 (10.6)	583 (61.8)	20 (2.1)	944 (100)

^a^Lower inner quadrant, lower outer quadrant, 6 o’clock

^b^3 and 9 o’clock

^c^Upper outer quadrant, upper inner quadrant, 12 o’clock

### Aesthetic Outcome

From 624 responders, 558 patients provided information about patient satisfaction with the aesthetic outcome (PRO). Results of PRO revealed a total of high degree of satisfaction with 78 % rating the aesthetic result as very good (55 %) or good (23 %). Combined with the rating “satisfactory” (10 %), a total of 88 % were satisfied with their surgical result. Five percent scored “fair,” 3 % “insufficient,” and 5 % “unsatisfactory.”

### Aesthetic Outcome in Questionnaires and Breast Cancer Treatment Outcome Scale

We compared the ratings of aesthetic outcome in our customized questionnaires with the results of the Breast Cancer Treatment Outcome Scale (BCTOS).^12^ In the BCTOS ratings, patients evaluated the outcome of the treated vs. untreated breast in 80.3 and 83.5 %, respectively, as “no difference or almost no difference” regarding the size and form of the breast. We found a significant correlation for good functional and aesthetic outcome in the BCTOS (= no difference or almost no difference) with high ratings of satisfaction in our customized questionnaire (*p* < 0.001).

### Factors that Influence the Perception of the Aesthetic Result

Factors that negatively influence the assessment of the aesthetic result were postoperative pain, wound infection, and issues related to scars. A higher intensity of pain on a visual analogue scale (VAS) ≥5 also was associated with less satisfaction with aesthetic outcome (*p* < 0.0001). Vice versa, those patients rating the aesthetic result as “very good” and “good” experienced low pain intensity in 86.3 % during the period of 14 days after surgery. This trend corresponded well with the perception of patients beyond the first 2 weeks after surgery.

Complication rate was low with 3.3 % wound infection (20/624), 9.5 % broadening of the scars (56/624), and 7.9 % occurrence of keloids (47/624). A total of 60.1 % of patients experienced perceptibility of the scar by palpation (346/624). The correlation of these complications with a lower rating of the aesthetic result was statistically significant [wound infection (*p* < 0.0001), broadening of the scars (*p* < 0.0001), perceptibility of the scars by palpation (*p* < 0.0001), and occurrence of keloids (*p* < 0.0001)].

#### Factors That Do Not Influence the Rating of the Aesthetic Result

The following factors did not exert any impact on the patient satisfaction (*p* > 0.05):

- Resection volume
- Type of oncoplastic technique
- Age at time of surgery
- Body mass index (BMI) at time of surgery
- Tumor localization

**Fig. 3:**
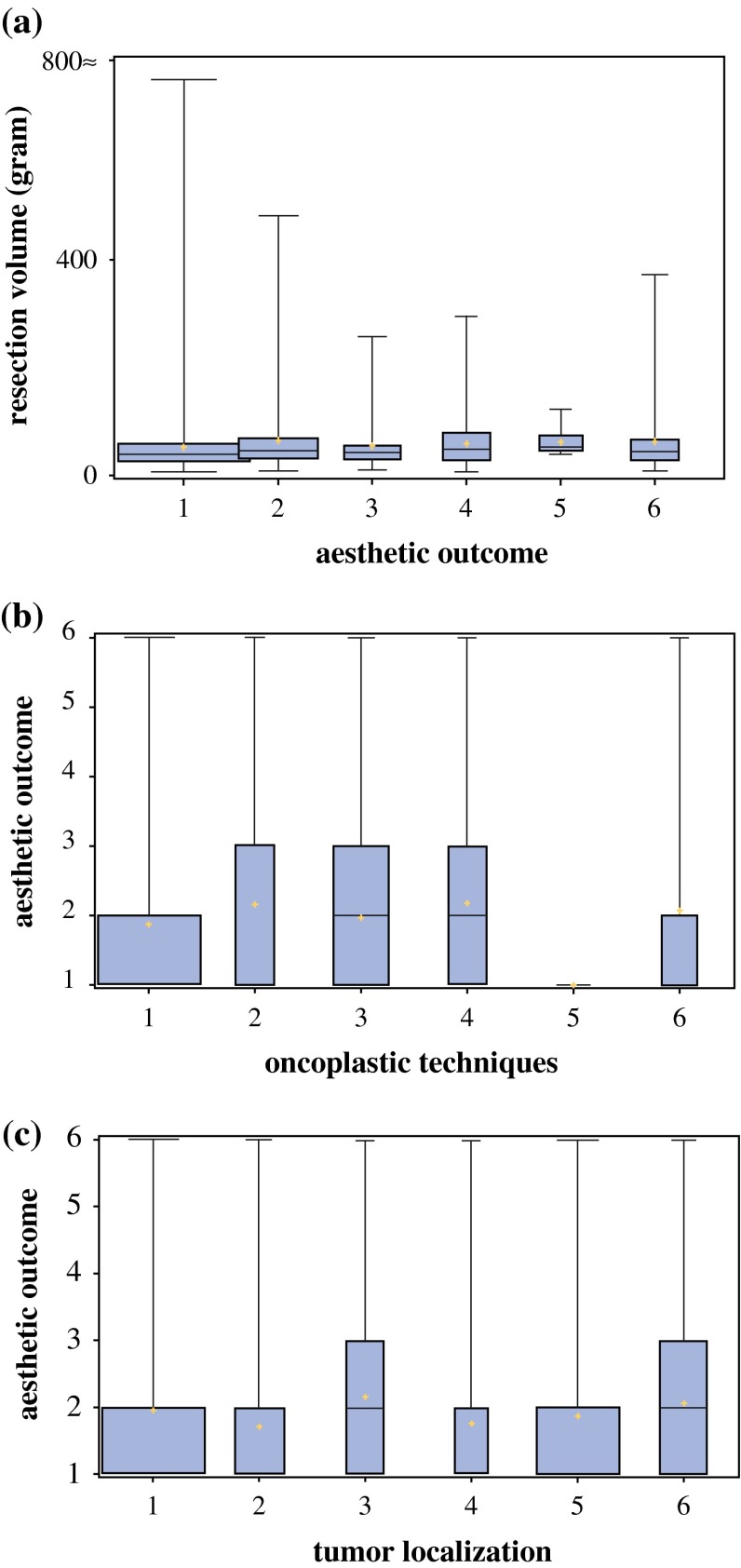
**a** Aesthetic outcome (1 = very good; 2 = good; 3 = satisfactory; 4 = fair; 5 = insufficient; 6 = unsatisfactory) and the influence of resection volume (g). **b** Aesthetic outcome (1 = very good; 2 = good; 3 = satisfactory; 4 = fair; 5 = insufficient; 6 = unsatisfactory) and the influence of the choice of the primary surgical technique (1 = glandular rotation mammoplasty; 2 = dermoglandular rotation mammoplasty; 3 = tumor-adapted mastopexies; 4 = lateral thoracic advancement flap; 5 = latissimus-dorsi-flap; 6 = others). **c** Aesthetic outcome (1 = very good; 2 = good; 3 = satisfactory; 4 = fair; 5 = insufficient; 6 = unsatisfactory) and the influence of the tumor localization (1 = upper outer; 2 = upper inner; 3 = lower outer; 4 = lower inner; 5 = transition; 6 = multicentric and multifocal)

The following boxplots illustrate the independence of the aesthetic result from the factors: resection volume, type of surgery, and localization of the tumor (Fig. 3a–c).

Patients across all ages and all BMI groups reported a high degree of satisfaction with the aesthetic result. BMI did not have any impact on the aesthetic result (*p* > 0.05). The majority of patients denoted that oncoplastic surgery *did not* have an impact on partnership (88 %) or body image (74 %).

### Reexcision Rate and Necessity of Secondary Mastectomy to Achieve Local Control

In 11.4 % (108/944) of patients, margins were unclear after first oncoplastic surgery, of which 89.8 % (97/108) underwent reexcision. This resulted in a margin clearance of 96.9 % for all patients opting for reexcision. However 10.2 % (11/108) of patients did not undergo reexcision. Finally, a proportion of 1.5 % (14/944) of the whole oncoplastic cohort remained with unclear margins.

Factors that influenced the clearness of margins were multicentricity or multifocality of tumors (*p* < 0.001). Neither *T* stage nor resection volume had an impact on primarily achieved margin status (*p* > 0.05).

In a total of 7.2 % (68/944) of the cohort, a secondary mastectomy had to be performed. We identified ductal carcinoma in situ (DCIS) (*p* = 0.001) *with and without* invasive subtype as an independent risk factor for a subsequent mastectomy, and likewise this was the case for lobular histology (*p* = 0.001). A total of 13.6 % (*n* = 15/110) of lobular histology underwent mastectomy, whereas 5.8 % (*n* = 33/572) of invasive ductal histological subtype underwent this procedure.

Mastectomy as a subsequent procedure did not correlate with the choice of oncoplastic technique used for primary surgery (*p* > 0.05).

In 77.4 % (*n* = 731/944) of patients, the first oncoplastic operation was the definitive and final procedure. In 22.6 % (213/944) of cases, patients underwent two more surgical procedures. Reasons other than clearance of margins for a subsequent operation were bleeding in 5.0 % (47/944), contralateral alignment in 1.9 % (18/944), and dehiscence of scars in 0.4 % (4/944).

### Local Recurrence Rates

Thirty-eight women (4.0 %) experienced a local recurrence at a median follow-up time of 5.2 years. We detected no significant difference between the oncoplastic techniques. Five-year disease-free survival was 90.9 %, and 5-year overall survival was 94.5 %.

## Discussion

Optimal local tumor control and the prevention of recurrence or metastatic spread by surgery, radiotherapy, and systemic therapy are the primary goal of breast cancer treatment.^14^ The systematization of oncoplastic operations presented in our study facilitates a high degree of local oncological control for any tumor localization and almost any tumor size. Nomograms for oncoplastic surgery were published by Veronesi et al., who presented reconstructive variations after quadrantectomy with higher aesthetic outcomes.^15^ The local recurrence rate in our study of 4.0 % is low in the context of international literature, where recurrence rates up to 9 % are reported in similar cohorts.^16^,^17^ In a recent meta-analysis of Losken et al., local recurrence occurred in oncoplastic patients only at a rate of 4 % compared with patients operated with breast-conserving therapy (7 %).^1^ The rate of subsequent operations performed in our cohort corresponds with international data, in which reexcision rates from 10 to 18 % are described.^18^–^20^ The conversion rate from oncoplastic procedure to mastectomy is low at 7.2 % in our cohort. This emphasizes the fact that extensive autologous or heterologous reconstructions may be spared when appropriate oncoplastic techniques are primarily applied.^21^ DCIS, multicentricity, and multifocality are known factors for a higher rate of local recurrence and consecutively mastectomy.^14^ Multicentric DCIS has been described as an indication for a subsequent mastectomy.^22^

Clear margins go along with a reduced risk of local recurrence whatever the distance of margins has been.^23^ In international literature, rates of unclear margins from 10.6 to 38 % are described.^24^–^28^ Our results were comparably low with a rate of 11.4 % of unclear margins after primary surgery.

Only a few patients of our cohort refused reexcision of unclear margins (*n* = 11). We did not detect any recurrences in these patients during the period of 5-year follow-up.

There was no significant correlation between a certain oncoplastic technique and the rate of unclear margins. In 2013, Down et al. demonstrated an advantage of oncological safety (lower rate of unclear margins) through oncoplastic techniques in a cohort of 158 patients. Oncoplastic techniques have been applied whenever the estimated volume of resection was higher than 10 % of breast volume in the inner quadrants and 20 % in the outer quadrants.^29^ Similar recommendations were given by Veronesi et al.^30^

Not only oncological safety but also aesthetic aspects are centrally incorporated in the oncoplastic concept as Cardoso and Heneghan et al. stated.^31^,^32^ Controversial data are reported as to patient satisfaction with the aesthetic result with a range of 40–89.5 %.^1^,^33^ We recorded a high degree of patient satisfaction with the aesthetic result at the upper range of internationally published data with 88 % of patients being satisfied with the aesthetic result.

Over a wide range of 11–793 (median 32) g, breast conservation appears feasible following this nomogram. Breast-tumor ratio and relative excision volume goes along with generally worse cosmetic outcome if conventional breast-conserving therapies are applied.^34^–^37^ Yang et al. demonstrated a high degree of satisfaction with aesthetic results independent of the extent of excision volume by using oncoplastic surgical techniques.^7^ Waljee et al. reported as treatment-related factors predictive for asymmetry: reexcision, postoperative seroma, and radiotherapy.^38^ These factors were comparatively low in our cohort. Tumor localizations in the upper inner and lower outer quadrant impose a high challenge to the surgeon´s skills with the risk of asymmetry.^39^ Even in difficult tumors locations, we did not find a deterioration of patient satisfaction; likewise, it was published in a smaller case series by Fitoussi et al.^40^ Most recently, smaller studies with oncoplastic techniques reported 72 patients that underlined the necessity of contralateral alignment during the same operation, which was performed in 53 of 72 patients (73.6 %) published by Rose et al.^20^ We report a low rate of 1.9 % (18/944 patients) with contralateral alignment operation. BMI and age did not have a negative impact on the rating of the aesthetic outcome in our cohort contrary to other study data.^31^,^41^

## Conclusions

The systematization of oncoplastic techniques in the concept presented in this study (Fig. 3) embedded in a multimodal concept of breast cancer therapy^1^–^3^ facilitates tumor control with the use of uncomplicated techniques adapted to tumor site and size with a median resection of 32 (range 11–793) g in this cohort. It demonstrated a high level of satisfaction of patient-reported outcome using this concept. These favourable results were independent from age, BMI, resection volume, tumor localization, and type of oncoplastic surgery. We identified postoperative *pain* as an important factor to deteriorate patient satisfaction. This underlines the need for a well-structured pain management schedule postoperatively to eliminate this negative factor that influences patient assessment of the surgical result.

## Electronic supplementary material

Below is the link to the electronic supplementary material.

Supplementary material 1 (DOCX 49 kb)

Supplementary material 2 (DOCX 16 kb)
