# Supplementary figures and images for: Systematization of Oncoplastic Surgery: Selection of Surgical Techniques and Patient-Reported Outcome in a Cohort of 1,035 Patients
Source: Ann Surg Oncol. 2015 Feb 12;22(11):3730–7. doi: 10.1245/s10434-015-4396-4 (PMC4565865; doi:10.1245/s10434-015-4396-4)

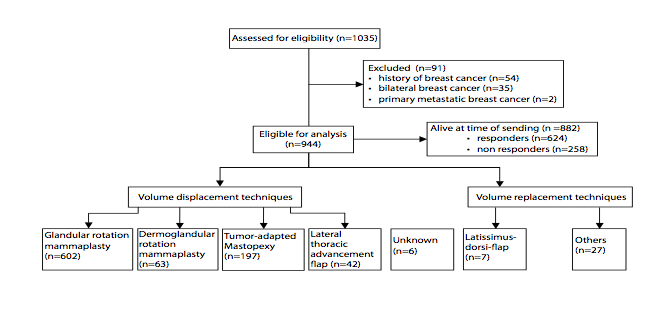


**Supplement. Material 1: REMARK-DIAGRAMM**

Supplement: Supplementary file 1 — Supplementary material 1 (DOCX 49 kb) [file 10434_2015_4396_MOESM1_ESM.docx]
