# Supplementary material for: Systematization of Oncoplastic Surgery: Selection of Surgical Techniques and Patient-Reported Outcome in a Cohort of 1,035 Patients
Source: Ann Surg Oncol. 2015 Feb 12;22(11):3730–7. doi: 10.1245/s10434-015-4396-4 (PMC4565865; doi:10.1245/s10434-015-4396-4)
Supplement: Supplementary file 2 — Supplementary material 2 (DOCX 16 kb) [file 10434_2015_4396_MOESM2_ESM.docx]

**Tumor characteristics** **are displayed in the** **supplement. material 2**.

| **Supplement.Material 2 : Tumor characteristics** | secondary mastectomy (n=68) | | cohort  (n=944) | | responders (n=624) | | |
| --- | --- | --- | --- | --- | --- | --- | --- |
| Characteristic | No. | % | No. | % | No. | % | |
| Histological type: non-invasive | 17 | 15.5 | 110 | 11.7 | 70 | 11.2 | |
| DCIS | 17 | 16.2 | 105 | 11.1 | 67 | 10.7 | |
| CLIS | 0 | 0 | 3 | 0.3 | 2 | 0.3 | |
| non-invasive, subtype unknown | 0 | 0 | 2 | 0.2 | 1 | 0.2 | |
| invasive | 51 | 6.3 | 811 | 85.9 | 542 | 86.9 | |
| invasive-ductal | 33 | 5.8 | 572 | 60.6 | 378 | 60.7 | |
| invasive-lobular | 15 | 13.6 | 110 | 11.6 | 74 | 11.9 | |
| „others“ | 2 | 2.2 | 89 | 9.4 | 63 | 10.1 | |
| invasive, subtype unknown | 1 | 2.5 | 40 | 4.2 | 26 | 4.2 | |
| histological type completely unknown | 0 | 0.0 | 23 | 2.4 | 12 | 1.9 | |
| Histological group | | | | | | |  |
| invasive | 14 | 2.7 | 518 | 54.9 | 348 | 55.8 | |
| non-invasive | 17 | 15.5 | 110 | 11.7 | 70 | 11.2 | |
| both (invasive and non-invasive) | 37 | 12.6 | 293 | 31.0 | 194 | 31.1 | |
| unknown | 0 | 0.0 | 23 | 2.4 | 12 | 1.9 | |
| Tumor localization | | | | | | |  |
| upper outer quadrant | 15 | 4.4 | 344 | 36.5 | 243 | 38.9 | |
| upper inner quadrant | 2 | 1.7 | 116 | 12.3 | 70 | 11.2 | |
| inferior outer quadrant | 2 | 3.1 | 65 | 6.9 | 38 | 6.1 | |
| inferior inner quadrant | 1 | 2.9 | 34 | 3.6 | 23 | 3.7 | |
| 12 o`clock | 5 | 4.1 | 123 | 13.0 | 82 | 13.1 | |
| 3 o`clock | 4 | 8.5 | 47 | 5.0 | 30 | 4.9 | |
| 6 o`clock | 0 | 0 | 31 | 3.3 | 20 | 3.2 | |
| 9 o`clock | 2 | 6.3 | 32 | 3.4 | 21 | 3.4 | |
| retromamillar | 1 | 4.8 | 21 | 2.2 | 13 | 2.1 | |
| multicentric | 7 | 18.9 | 37 | 3.9 | 20 | 3.2 | |
| multifocal | 27 | 36.5 | 74 | 7.8 | 50 | 8.0 | |
| unknown | 2 | 1 | 20 | 2.1 | 14 | 2.2 | |
| Intrinsic subtype | | | | | | |  |
| Luminal A | 42 | 7.1 | 591 | 62.6 | 400 | 64.1 | |
| Luminal B/Her2 negative | 6 | 8.2 | 73 | 7.7 | 53 | 8.5 | |
| Luminal B/Her2 positive | 7 | 8.7 | 80 | 8.5 | 53 | 8.5 | |
| Her2 positive non-luminal | 7 | 13.0 | 54 | 5.7 | 24 | 3.8 | |
| triple negative ductal | 5 | 5.2 | 97 | 10.3 | 63 | 10.1 | |
| unknown | 1 | 2.0 | 49 | 5.2 | 31 | 5.0 | |
| Oncoplastic technique | | | | | | |  |
| Glandular rotation mammaplasty | 46 | 7.6 | 602 | 63.8 | 413 | 66.2 | |
| Dermoglandular rotation mammaplasty | 3 | 4.8 | 63 | 6.7 | 33 | 5.3 | |
| Tumor-adapted mastopexy | 12 | 6.1 | 197 | 20.9 | 124 | 19.9 | |
| Thoracal advancement flap | 5 | 11.9 | 42 | 4.5 | 32 | 5.1 | |
| Latissimus-dorsi flap | 0 | 0.0 | 7 | 0.7 | 4 | 0.6 | |
| others | 2 | 7.4 | 29 | 2.9 | 15 | 2.4 | |
| unknown | 0 | 0.0 | 6 | 0.6 | 3 | 0.5 | |
